# [*rac*-2-(1-Amino­eth­yl)phenyl-κ^2^ *C* ^1^,*N*](ethyl­endiamine-κ^2^ *N*,*N*′)palladium(II) 3,5-dimethyl­benzoate

**DOI:** 10.1107/S1600536810028369

**Published:** 2010-07-21

**Authors:** Mihaela-Diana Şerb, Irmgard Kalf, Ulli Englert

**Affiliations:** a Department of Inorganic Chemistry, Faculty of Applied Chemistry and Materials Science, University Politehnica of Bucharest, Polizu 1, RO-011061 Bucharest, Romania; b Institut für Anorganische Chemie, RWTH Aachen University, Landoltweg 1, 52074 Aachen, Germany

## Abstract

In the title compound, [Pd(C_8_H_10_N)(C_2_H_8_N_2_)](C_9_H_9_O_2_), the palladium ion is coordinated in a distorted square-planar fashion by the two N atoms from the chelating ethyl­enediamine group and by the N and a C atom of the deprotonated chiral amine. The resulting cationic complex and the 3,5-dimethyl­benzoate anion are inter­connected by N—H⋯O hydrogen bonds.

## Related literature

For related organopalladium complexes with chelating oxygen donor ligands, see: Calmuschi & Englert (2002 ▶, 2005*a* ▶,*b* ▶,*c* ▶); Calmuschi *et al.* (2004 ▶). For related organopalladium complexes with nitro­gen donor ligands, see: Kalf *et al.* (2006 ▶, 2008 ▶); Şerb *et al.* (2010 ▶). For hydrogen-bond motifs, see: Etter *et al.* (1990 ▶); Etter (1991 ▶).
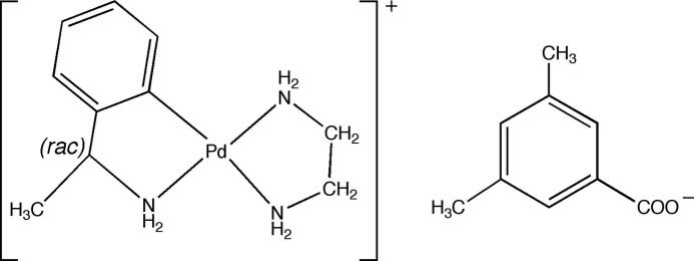


## Experimental

### 

#### Crystal data

[Pd(C_8_H_10_N)(C_2_H_8_N_2_)](C_9_H_9_O_2_)
*M* *_r_* = 435.84
Monoclinic, 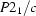

*a* = 7.9624 (9) Å
*b* = 28.615 (3) Å
*c* = 8.5964 (10) Å
β = 100.616 (2)°
*V* = 1925.1 (4) Å^3^
*Z* = 4
Mo *K*α radiation
μ = 0.98 mm^−1^
*T* = 110 K
0.19 × 0.17 × 0.03 mm

#### Data collection

Bruker SMART CCD area-detector diffractometer
Absorption correction: multi-scan (*MULABS*; Blessing, 1995 ▶; Spek, 2009 ▶) *T*_min_ = 0.836, *T*_max_ = 0.971
17762 measured reflections
4375 independent reflections
3310 reflections with *I* > 2σ(*I*)
*R*_int_ = 0.076

#### Refinement

*R*[*F* ^2^ > 2σ(*F* ^2^)] = 0.048
*wR*(*F* ^2^) = 0.092
*S* = 0.98
4375 reflections
229 parameters
H-atom parameters constrained
Δρ_max_ = 0.73 e Å^−3^
Δρ_min_ = −1.65 e Å^−3^

### 

Data collection: *SMART* (Bruker, 2001 ▶); cell refinement: *SAINT-Plus* (Bruker, 1999 ▶); data reduction: *SAINT-Plus*; program(s) used to solve structure: *SHELXS97* (Sheldrick, 2008 ▶); program(s) used to refine structure: *SHELXL97* (Sheldrick, 2008 ▶); molecular graphics: *PLATON* (Spek, 2009 ▶); software used to prepare material for publication: *SHELXL97* .

## Supplementary Material

Crystal structure: contains datablocks global, I. DOI: 10.1107/S1600536810028369/bt5293sup1.cif

Structure factors: contains datablocks I. DOI: 10.1107/S1600536810028369/bt5293Isup2.hkl

Additional supplementary materials:  crystallographic information; 3D view; checkCIF report

## Figures and Tables

**Table:** 

Pd1—N1	1.910 (3)
Pd1—N3	1.941 (3)
Pd1—C3	2.000 (4)
Pd1—N2	2.119 (3)

**Table:** 

N1—Pd1—N3	178.30 (14)
N1—Pd1—C3	79.44 (16)
N3—Pd1—C3	102.15 (15)
N1—Pd1—N2	98.49 (14)
N3—Pd1—N2	79.89 (13)
C3—Pd1—N2	176.25 (16)

**Table 2:** Hydrogen-bond geometry (Å, °)

*D*—H⋯*A*	*D*—H	H⋯*A*	*D*⋯*A*	*D*—H⋯*A*
N1—H1*A*⋯O1	0.92	2.18	3.082 (5)	167
N1—H1*B*⋯O1^i^	0.92	2.08	2.946 (5)	156
N2—H2*A*⋯O1^i^	0.92	2.06	2.892 (4)	151
N3—H3*A*⋯O2^ii^	0.92	2.03	2.937 (5)	168
N3—H3*B*⋯O2^iii^	0.92	2.38	3.098 (4)	135

Symmetry codes: (i) 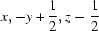; (ii) 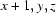; (iii) 
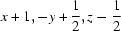
.

**Fig. 1.**
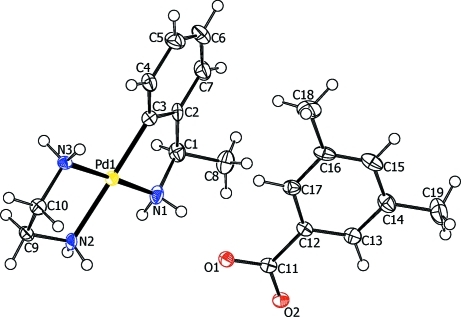
: PLATON (Spek, 2009) plot with displacement ellipsoids at 50% probability; H atoms are represented by spheres of arbitrary radius.

**Fig. 2.**
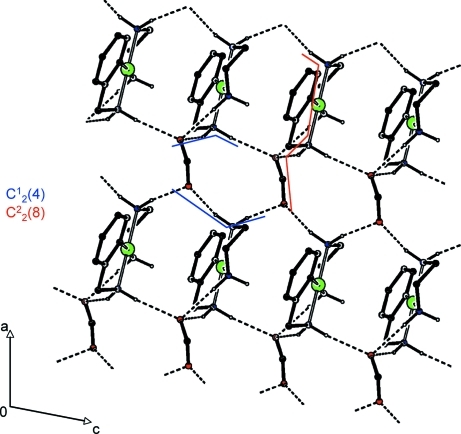
: Hydrogen-bond motifs. The 3,5-dimethylphenyl group of the anion, the methyl group attached to the cation and H atoms attached to carbon have been omitted for clarity.

**Fig. 3.**
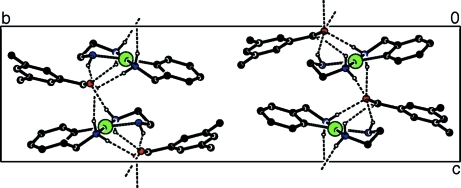
: Packing diagram of the title compound. The dashed lines indicate the hydrogen bonds. H atoms not involved in H bonding have been omitted for clarity.

## supplementary crystallographic information

### Comment

The complex cation (Fig. 1) is essentially square planar: the distance of the metal center to the least-squares plane through the coordinating atoms amounts to 0.0331 (3) Å. The bond lengths between palladium and ethylenediamine nitrogen atoms differ significantly: The Pd—N distance *trans* to carbon is 2.119 (3) Å and hence longer than the bond to the N donor atom *trans* to the amino group (1.941 (3) Å) (Table 1). This observation is in agreement with the distance pattern observed for related organopalladium complexes with chelating oxygen donor ligands (Calmuschi & Englert, 2002; Calmuschi *et al.*, 2004; Calmuschi & Englert, 2005*a*; Calmuschi & Englert, 2005*b*; Calmuschi & Englert, 2005*c*) and nitrogen donor ligands (Kalf *et al.*, 2006; Kalf *et al.*, 2008). The structure is extended through moderately strong N—H···O hydrogen bonds to give rise to a two-dimensional network. With the exception of H2b (attached to N2 of the ethylendiamine ligand) all potential H donors find an acceptor in reasonable geometry for hydrogen bonding. The intermolecular motifs in the *a* direction are C_2_^2^(8); in the *c* direction C_2_^1^(4) motifs can be observed.(Etter *et al.*, 1990; Etter,   1991) (Fig. 2). The hydrogen bond parameters are presented in Table 2. The flat cationic complexes form stacks extending in the *c* direction; the shortest Pd···Pd separation amounts to 4.4819 (7) Å. Figure 3 shows the packing diagram of the title compound. The molecular volume of the title compound (calculated as V/*Z*) is very similar to the molecular volume of {(*rac*)-[2-(1-aminoethyl)phenyl-κ^2^-*C^1^,N*] (ethylendiamine)palladium(II)} 3-methylbenzoate hydrate compound reported by Şerb *et al.*, (2010), in which the additional solvent water molecule compensates the smaller size of the anion.

### Experimental

46 mg (0.76 mmol) ethylenediamine are added to a solution of 200 mg (0.38 mmol) [{Pd(µ-Cl)(C_6_H_4_CH-MeNH_2_)}_2_] (Calmuschi & Englert, 2002) in 50 ml MeOH at 50 ° C. 196 mg (0.76 mmol) silver-3,5-dimethylbenzoate are added; the suspension is stirred for 30 min and allowed to cool to room temperature, and AgCl is removed by filtration. After evaporation of the solvent *in vacuo*, the product is obtained in almost quantitative yield. Slow evaporation of the solvent under ambient conditions gives crystals suitable for X-ray diffraction.

### Refinement

H atoms were introduced in their idealized positions with C_aryl_—H 0.95 Å, *U*_iso_(H) = 1.2*U*_eq_(C); C_methyl_—H 0.98 Å, *U*_iso_(H) = 1.5*U*_eq_(C); C_ethylene_—H 0.99 Å, *U*_iso_(H) = 1.2*U*_eq_(C) and N—H 0.92 Å, *U*_iso_(H) = 1.2*U*_eq_(N) and refined using a riding model.

### Crystal data

**Table:**

[Pd(C_8_H_10_N)(C_2_H_8_N_2_)](C_9_H_9_O_2_)	*F*(000) = 896
*M**_r_* = 435.84	*D*_x_ = 1.504 Mg m^−^^3^
Monoclinic, *P*2_1_/*c*	Mo *K*α radiation, λ = 0.71073 Å
Hall symbol: -P 2ybc	Cell parameters from 3595 reflections
*a* = 7.9624 (9) Å	θ = 2.4–24.6°
*b* = 28.615 (3) Å	µ = 0.98 mm^−^^1^
*c* = 8.5964 (10) Å	*T* = 110 K
β = 100.616 (2)°	Plate, yellow
*V* = 1925.1 (4) Å^3^	0.19 × 0.17 × 0.03 mm
*Z* = 4	

### Data collection

**Table:**

Bruker SMART CCD area-detector diffractometer	4375 independent reflections
Radiation source: fine-focus sealed tube	3310 reflections with *I* > 2σ(*I*)
graphite	*R*_int_ = 0.076
ω scans	θ_max_ = 27.5°, θ_min_ = 2.5°
Absorption correction: multi-scan (*MULABS*; Blessing, 1995; Spek, 2009)	*h* = −10→10
*T*_min_ = 0.836, *T*_max_ = 0.971	*k* = −37→33
17762 measured reflections	*l* = −10→10

### Refinement

**Table:**

Refinement on *F*^2^	Primary atom site location: structure-invariant direct methods
Least-squares matrix: full	Secondary atom site location: difference Fourier map
*R*[*F*^2^ > 2σ(*F*^2^)] = 0.048	Hydrogen site location: inferred from neighbouring sites
*wR*(*F*^2^) = 0.092	H-atom parameters constrained
*S* = 0.98	*w* = 1/[σ^2^(*F*_o_^2^) + (0.029*P*)^2^] where *P* = (*F*_o_^2^ + 2*F*_c_^2^)/3
4375 reflections	(Δ/σ)_max_ < 0.001
229 parameters	Δρ_max_ = 0.73 e Å^−^^3^
0 restraints	Δρ_min_ = −1.65 e Å^−^^3^

### Special details

**Table:**

Geometry. All e.s.d.'s (except the e.s.d. in the dihedral angle between two l.s. planes) are estimated using the full covariance matrix. The cell e.s.d.'s are taken into account individually in the estimation of e.s.d.'s in distances, angles and torsion angles; correlations between e.s.d.'s in cell parameters are only used when they are defined by crystal symmetry. An approximate (isotropic) treatment of cell e.s.d.'s is used for estimating e.s.d.'s involving l.s. planes.
Refinement. Refinement of *F*^2^ against ALL reflections. The weighted *R*-factor *wR* and goodness of fit *S* are based on *F*^2^, conventional *R*-factors *R* are based on *F*, with *F* set to zero for negative *F*^2^. The threshold expression of *F*^2^ > σ(*F*^2^) is used only for calculating *R*-factors(gt) *etc*. and is not relevant to the choice of reflections for refinement. *R*-factors based on *F*^2^ are statistically about twice as large as those based on *F*, and *R*- factors based on ALL data will be even larger.

### Fractional atomic coordinates and isotropic or equivalent isotropic displacement parameters (Å^2^)

**Table:**

	*x*	*y*	*z*	*U*_iso_*/*U*_eq_	
Pd1	0.94093 (4)	0.227805 (11)	0.25811 (4)	0.01652 (10)	
N1	0.7080 (4)	0.20820 (12)	0.2066 (5)	0.0234 (9)	
H1A	0.6686	0.2026	0.2990	0.028*	
H1B	0.6439	0.2321	0.1544	0.028*	
N2	0.8992 (4)	0.30017 (12)	0.2865 (4)	0.0203 (8)	
H2A	0.8093	0.3100	0.2105	0.024*	
H2B	0.8713	0.3054	0.3842	0.024*	
N3	1.1755 (4)	0.24963 (12)	0.3089 (4)	0.0160 (8)	
H3A	1.2379	0.2301	0.3827	0.019*	
H3B	1.2233	0.2489	0.2193	0.019*	
C1	0.6809 (5)	0.16412 (15)	0.1033 (6)	0.0252 (11)	
H1	0.6603	0.1733	−0.0109	0.030*	
C2	0.8333 (5)	0.13607 (15)	0.1385 (5)	0.0213 (10)	
C3	0.9712 (5)	0.15983 (14)	0.2170 (5)	0.0179 (9)	
C4	1.1143 (5)	0.13512 (15)	0.2544 (5)	0.0235 (11)	
H4	1.2141	0.1493	0.3131	0.028*	
C5	1.1188 (6)	0.08822 (16)	0.2077 (6)	0.0304 (12)	
H5	1.2232	0.0716	0.2361	0.036*	
C6	0.9840 (6)	0.06567 (17)	0.1253 (6)	0.0342 (13)	
H6	0.9911	0.0341	0.0929	0.041*	
C7	0.8403 (6)	0.08982 (16)	0.0915 (6)	0.0291 (12)	
H7	0.7404	0.0753	0.0343	0.035*	
C8	0.5346 (5)	0.13765 (17)	0.1359 (6)	0.0314 (12)	
H8A	0.5043	0.1134	0.0552	0.047*	
H8B	0.4372	0.1587	0.1338	0.047*	
H8C	0.5639	0.1231	0.2405	0.047*	
C9	1.0466 (5)	0.32579 (14)	0.2732 (5)	0.0202 (10)	
H9A	1.0405	0.3581	0.3133	0.024*	
H9B	1.0639	0.3270	0.1621	0.024*	
C10	1.1831 (5)	0.29881 (14)	0.3734 (5)	0.0203 (10)	
H10A	1.1655	0.2986	0.4845	0.024*	
H10B	1.2960	0.3129	0.3704	0.024*	
O1	0.6039 (3)	0.20443 (10)	0.5344 (4)	0.0207 (7)	
O2	0.3555 (3)	0.19477 (10)	0.5774 (4)	0.0217 (7)	
C11	0.4914 (5)	0.17924 (15)	0.5705 (5)	0.0173 (9)	
C12	0.5283 (5)	0.12878 (14)	0.6100 (5)	0.0169 (9)	
C13	0.4191 (5)	0.10026 (15)	0.6716 (5)	0.0221 (10)	
H13	0.3135	0.1132	0.6874	0.027*	
C14	0.4520 (6)	0.05353 (16)	0.7128 (6)	0.0261 (11)	
C15	0.5963 (6)	0.03531 (16)	0.6867 (6)	0.0279 (11)	
H15	0.6226	0.0035	0.7119	0.033*	
C16	0.7073 (5)	0.06234 (16)	0.6236 (6)	0.0244 (11)	
C17	0.6705 (5)	0.10894 (15)	0.5866 (5)	0.0214 (10)	
H17	0.7506	0.1270	0.5431	0.026*	
C18	0.8671 (6)	0.04280 (17)	0.6026 (7)	0.0401 (14)	
H18A	0.9516	0.0467	0.7002	0.060*	
H18B	0.9070	0.0589	0.5155	0.060*	
H18C	0.8523	0.0095	0.5778	0.060*	
C19	0.3363 (6)	0.02328 (17)	0.7852 (7)	0.0453 (15)	
H19A	0.3451	−0.0090	0.7495	0.068*	
H19B	0.2184	0.0342	0.7533	0.068*	
H19C	0.3690	0.0246	0.9008	0.068*	

### Atomic displacement parameters (Å^2^)

**Table:**

	*U*^11^	*U*^22^	*U*^33^	*U*^12^	*U*^13^	*U*^23^
Pd1	0.01214 (14)	0.01639 (18)	0.02123 (19)	0.00006 (14)	0.00361 (12)	0.00117 (17)
N1	0.0126 (17)	0.025 (2)	0.034 (2)	−0.0002 (15)	0.0080 (16)	0.0100 (19)
N2	0.0122 (16)	0.020 (2)	0.030 (2)	−0.0036 (14)	0.0081 (16)	0.0033 (17)
N3	0.0129 (16)	0.0155 (18)	0.020 (2)	0.0031 (14)	0.0037 (15)	−0.0011 (16)
C1	0.019 (2)	0.023 (3)	0.033 (3)	−0.0045 (19)	0.001 (2)	0.002 (2)
C2	0.020 (2)	0.017 (2)	0.029 (3)	−0.0051 (18)	0.010 (2)	0.004 (2)
C3	0.016 (2)	0.020 (2)	0.018 (2)	0.0017 (17)	0.0045 (17)	−0.0002 (19)
C4	0.020 (2)	0.020 (3)	0.030 (3)	−0.0040 (18)	0.004 (2)	0.002 (2)
C5	0.026 (2)	0.024 (3)	0.041 (3)	0.006 (2)	0.006 (2)	0.000 (2)
C6	0.038 (3)	0.017 (3)	0.049 (4)	−0.004 (2)	0.011 (3)	−0.005 (2)
C7	0.026 (2)	0.023 (3)	0.037 (3)	−0.005 (2)	0.004 (2)	0.001 (2)
C8	0.020 (2)	0.040 (3)	0.032 (3)	−0.009 (2)	−0.001 (2)	−0.002 (2)
C9	0.017 (2)	0.014 (2)	0.030 (3)	−0.0012 (17)	0.0064 (19)	−0.001 (2)
C10	0.015 (2)	0.022 (3)	0.023 (3)	0.0003 (17)	0.0002 (18)	−0.001 (2)
O1	0.0110 (13)	0.0182 (16)	0.033 (2)	−0.0024 (12)	0.0045 (13)	−0.0022 (14)
O2	0.0121 (14)	0.0216 (17)	0.032 (2)	0.0077 (12)	0.0053 (13)	0.0057 (14)
C11	0.0144 (19)	0.021 (2)	0.015 (2)	0.0016 (17)	−0.0022 (17)	−0.0028 (19)
C12	0.0145 (19)	0.015 (2)	0.019 (3)	0.0016 (16)	−0.0022 (17)	−0.0004 (19)
C13	0.013 (2)	0.024 (3)	0.029 (3)	−0.0003 (17)	0.0006 (19)	−0.005 (2)
C14	0.020 (2)	0.023 (3)	0.033 (3)	−0.0034 (19)	−0.001 (2)	−0.002 (2)
C15	0.029 (2)	0.018 (3)	0.032 (3)	0.006 (2)	−0.006 (2)	−0.004 (2)
C16	0.018 (2)	0.022 (3)	0.031 (3)	0.0084 (18)	−0.002 (2)	−0.006 (2)
C17	0.017 (2)	0.022 (3)	0.025 (3)	0.0001 (18)	0.0035 (19)	−0.006 (2)
C18	0.024 (3)	0.036 (3)	0.061 (4)	0.015 (2)	0.008 (3)	0.002 (3)
C19	0.030 (3)	0.033 (3)	0.072 (5)	−0.006 (2)	0.007 (3)	0.014 (3)

### Geometric parameters (Å, °)

**Table:**

Pd1—N1	1.910 (3)	C8—H8B	0.9800
Pd1—N3	1.941 (3)	C8—H8C	0.9800
Pd1—C3	2.000 (4)	C9—C10	1.475 (5)
Pd1—N2	2.119 (3)	C9—H9A	0.9900
N1—C1	1.535 (6)	C9—H9B	0.9900
N1—H1A	0.9200	C10—H10A	0.9900
N1—H1B	0.9200	C10—H10B	0.9900
N2—C9	1.406 (5)	O1—C11	1.233 (5)
N2—H2A	0.9200	O2—C11	1.181 (4)
N2—H2B	0.9200	C11—C12	1.500 (6)
N3—C10	1.510 (5)	C12—C17	1.315 (5)
N3—H3A	0.9200	C12—C13	1.368 (6)
N3—H3B	0.9200	C13—C14	1.396 (6)
C1—C2	1.440 (6)	C13—H13	0.9500
C1—C8	1.459 (6)	C14—C15	1.318 (6)
C1—H1	1.0000	C14—C19	1.482 (6)
C2—C3	1.360 (6)	C15—C16	1.361 (6)
C2—C7	1.388 (6)	C15—H15	0.9500
C3—C4	1.329 (5)	C16—C17	1.389 (6)
C4—C5	1.403 (6)	C16—C18	1.432 (6)
C4—H4	0.9500	C17—H17	0.9500
C5—C6	1.338 (6)	C18—H18A	0.9800
C5—H5	0.9500	C18—H18B	0.9800
C6—C7	1.323 (6)	C18—H18C	0.9800
C6—H6	0.9500	C19—H19A	0.9800
C7—H7	0.9500	C19—H19B	0.9800
C8—H8A	0.9800	C19—H19C	0.9800
			
N1—Pd1—N3	178.30 (14)	H8A—C8—H8B	109.5
N1—Pd1—C3	79.44 (16)	C1—C8—H8C	109.5
N3—Pd1—C3	102.15 (15)	H8A—C8—H8C	109.5
N1—Pd1—N2	98.49 (14)	H8B—C8—H8C	109.5
N3—Pd1—N2	79.89 (13)	N2—C9—C10	102.5 (3)
C3—Pd1—N2	176.25 (16)	N2—C9—H9A	111.3
C1—N1—Pd1	113.7 (2)	C10—C9—H9A	111.3
C1—N1—H1A	108.8	N2—C9—H9B	111.3
Pd1—N1—H1A	108.8	C10—C9—H9B	111.3
C1—N1—H1B	108.8	H9A—C9—H9B	109.2
Pd1—N1—H1B	108.8	C9—C10—N3	107.3 (3)
H1A—N1—H1B	107.7	C9—C10—H10A	110.2
C9—N2—Pd1	110.4 (3)	N3—C10—H10A	110.2
C9—N2—H2A	109.6	C9—C10—H10B	110.2
Pd1—N2—H2A	109.6	N3—C10—H10B	110.2
C9—N2—H2B	109.6	H10A—C10—H10B	108.5
Pd1—N2—H2B	109.6	O2—C11—O1	120.5 (4)
H2A—N2—H2B	108.1	O2—C11—C12	119.6 (4)
C10—N3—Pd1	110.7 (2)	O1—C11—C12	119.9 (4)
C10—N3—H3A	109.5	C17—C12—C13	115.4 (4)
Pd1—N3—H3A	109.5	C17—C12—C11	121.3 (4)
C10—N3—H3B	109.5	C13—C12—C11	123.3 (4)
Pd1—N3—H3B	109.5	C12—C13—C14	124.5 (4)
H3A—N3—H3B	108.1	C12—C13—H13	117.7
C2—C1—C8	110.0 (4)	C14—C13—H13	117.7
C2—C1—N1	108.2 (4)	C15—C14—C13	117.7 (4)
C8—C1—N1	110.3 (4)	C15—C14—C19	118.0 (5)
C2—C1—H1	109.4	C13—C14—C19	124.3 (4)
C8—C1—H1	109.4	C14—C15—C16	119.6 (4)
N1—C1—H1	109.4	C14—C15—H15	120.2
C3—C2—C7	123.3 (4)	C16—C15—H15	120.2
C3—C2—C1	113.4 (4)	C15—C16—C17	120.8 (4)
C7—C2—C1	123.2 (4)	C15—C16—C18	119.1 (4)
C4—C3—C2	115.6 (4)	C17—C16—C18	120.1 (4)
C4—C3—Pd1	126.9 (3)	C12—C17—C16	122.0 (4)
C2—C3—Pd1	117.5 (3)	C12—C17—H17	119.0
C3—C4—C5	120.3 (4)	C16—C17—H17	119.0
C3—C4—H4	119.8	C16—C18—H18A	109.5
C5—C4—H4	119.8	C16—C18—H18B	109.5
C6—C5—C4	123.6 (4)	H18A—C18—H18B	109.5
C6—C5—H5	118.2	C16—C18—H18C	109.5
C4—C5—H5	118.2	H18A—C18—H18C	109.5
C7—C6—C5	116.2 (5)	H18B—C18—H18C	109.5
C7—C6—H6	121.9	C14—C19—H19A	109.5
C5—C6—H6	121.9	C14—C19—H19B	109.5
C6—C7—C2	120.9 (4)	H19A—C19—H19B	109.5
C6—C7—H7	119.6	C14—C19—H19C	109.5
C2—C7—H7	119.6	H19A—C19—H19C	109.5
C1—C8—H8A	109.5	H19B—C19—H19C	109.5
C1—C8—H8B	109.5		
			
N3—Pd1—N1—C1	134 (5)	Pd1—C3—C4—C5	−175.4 (3)
C3—Pd1—N1—C1	−24.5 (3)	C3—C4—C5—C6	−0.2 (8)
N2—Pd1—N1—C1	152.4 (3)	C4—C5—C6—C7	−1.5 (8)
N1—Pd1—N2—C9	−160.2 (3)	C5—C6—C7—C2	0.7 (8)
N3—Pd1—N2—C9	19.2 (3)	C3—C2—C7—C6	1.8 (8)
C3—Pd1—N2—C9	−104 (2)	C1—C2—C7—C6	179.3 (5)
N1—Pd1—N3—C10	32 (5)	Pd1—N2—C9—C10	−46.1 (4)
C3—Pd1—N3—C10	−169.6 (3)	N2—C9—C10—N3	57.7 (4)
N2—Pd1—N3—C10	13.6 (3)	Pd1—N3—C10—C9	−43.9 (4)
Pd1—N1—C1—C2	29.6 (4)	O2—C11—C12—C17	172.2 (4)
Pd1—N1—C1—C8	150.0 (3)	O1—C11—C12—C17	−9.3 (6)
C8—C1—C2—C3	−136.7 (4)	O2—C11—C12—C13	−7.6 (7)
N1—C1—C2—C3	−16.1 (5)	O1—C11—C12—C13	170.8 (4)
C8—C1—C2—C7	45.6 (6)	C17—C12—C13—C14	1.7 (7)
N1—C1—C2—C7	166.2 (4)	C11—C12—C13—C14	−178.5 (4)
C7—C2—C3—C4	−3.4 (7)	C12—C13—C14—C15	−1.9 (7)
C1—C2—C3—C4	178.9 (4)	C12—C13—C14—C19	177.5 (5)
C7—C2—C3—Pd1	174.8 (4)	C13—C14—C15—C16	0.8 (7)
C1—C2—C3—Pd1	−2.9 (5)	C19—C14—C15—C16	−178.6 (5)
N1—Pd1—C3—C4	−165.9 (4)	C14—C15—C16—C17	0.2 (7)
N3—Pd1—C3—C4	14.7 (4)	C14—C15—C16—C18	177.1 (5)
N2—Pd1—C3—C4	137 (2)	C13—C12—C17—C16	−0.6 (7)
N1—Pd1—C3—C2	16.1 (3)	C11—C12—C17—C16	179.5 (4)
N3—Pd1—C3—C2	−163.2 (3)	C15—C16—C17—C12	−0.3 (7)
N2—Pd1—C3—C2	−41 (2)	C18—C16—C17—C12	−177.2 (5)
C2—C3—C4—C5	2.6 (7)		

### Hydrogen-bond geometry (Å, °)

**Table:**

*D*—H···*A*	*D*—H	H···*A*	*D*···*A*	*D*—H···*A*
N1—H1A···O1	0.92	2.18	3.082 (5)	167
N1—H1B···O1^i^	0.92	2.08	2.946 (5)	156
N2—H2A···O1^i^	0.92	2.06	2.892 (4)	151
N3—H3A···O2^ii^	0.92	2.03	2.937 (5)	168
N3—H3B···O2^iii^	0.92	2.38	3.098 (4)	135

Symmetry codes: (i) *x*, −*y*+1/2, *z*−1/2; (ii) *x*+1, *y*, *z*; (iii) *x*+1, −*y*+1/2, *z*−1/2.
